# Administrative Algorithms to identify Avascular necrosis of bone among patients undergoing upper or lower extremity magnetic resonance imaging: a validation study

**DOI:** 10.1186/s12891-017-1626-x

**Published:** 2017-06-19

**Authors:** Medha Barbhaiya, Yan Dong, Jeffrey A. Sparks, Elena Losina, Karen H. Costenbader, Jeffrey N. Katz

**Affiliations:** 1 Division of Rheumatology, Immunology and Allergy, Department of Medicine, Brigham and Women’s Hospital, Harvard Medical School, 60 Fenwood Avenue, Boston, MA 02115 USA; 2 OPKO Diagnostics, Woburn, MA USA; 3 Orthopedic and Arthritis Center for Outcomes Research, Department of Orthopedic Surgery, Brigham and Women’s Hospital, Harvard Medical School, Boston, MA USA

**Keywords:** Avascular necrosis, Avn, Osteonecrosis, Epidemiology, Magnetic, Resonance Imaging, MRI, administrative data, validation

## Abstract

**Background:**

Studies of the epidemiology and outcomes of avascular necrosis (AVN) require accurate case-finding methods. The aim of this study was to evaluate performance characteristics of a claims-based algorithm designed to identify AVN cases in administrative data.

**Methods:**

Using a centralized patient registry from a US academic medical center, we identified all adults aged ≥18 years who underwent magnetic resonance imaging (MRI) of an upper/lower extremity joint during the 1.5 year study period. A radiologist report confirming AVN on MRI served as the gold standard. We examined the sensitivity, specificity, positive predictive value (PPV) and positive likelihood ratio (LR^+^) of four algorithms (A-D) using International Classification of Diseases, 9th edition (ICD-9) codes for AVN. The algorithms ranged from least stringent (Algorithm A, requiring ≥1 ICD-9 code for AVN [733.4X]) to most stringent (Algorithm D, requiring ≥3 ICD-9 codes, each at least 30 days apart).

**Results:**

Among 8200 patients who underwent MRI, 83 (1.0% [95% CI 0.78–1.22]) had AVN by gold standard. Algorithm A yielded the highest sensitivity (81.9%, 95% CI 72.0–89.5), with PPV of 66.0% (95% CI 56.0–75.1). The PPV of algorithm D increased to 82.2% (95% CI 67.9–92.0), although sensitivity decreased to 44.6% (95% CI 33.7–55.9). All four algorithms had specificities >99%.

**Conclusion:**

An algorithm that uses a single billing code to screen for AVN among those who had MRI has the highest sensitivity and is best suited for studies in which further medical record review confirming AVN is feasible. Algorithms using multiple billing codes are recommended for use in administrative databases when further AVN validation is not feasible.

## Background

Avascular necrosis (AVN) of the bone is a debilitating and potentially devastating condition with approximately 20,000 to 30,000 new cases diagnosed yearly in the US [1, 2]. Ten percent of the over 400,000 total hip arthroplasties performed in the United States each year are due to symptomatic hip AVN [2, 3]. Although its pathogenesis is poorly understood, AVN results in ischemia, bone infarction, bone collapse, and joint destruction [4]. Magnetic resonance imaging (MRI) is the gold standard for detecting the earliest stages of AVN, with >99% specificity and sensitivity when compared against histologic examination or subsequent imaging [2, 5, 6].

Studies related to identifying risk factors, diagnostic tools, and management options for AVN require accurate case-finding methods. Administrative databases may be especially useful to estimate the incidence, prevalence and risk factors for AVN, a relatively rare disease [7]. However, the accuracy of administrative data in identifying disease varies across rheumatologic and orthopedic conditions [8]. The use of International Classification of Diseases, Ninth Revision (ICD-9)-based algorithms for the diagnosis of incident AVN has been previously assessed in a Boston Veterans Affair (VA) cohort; this study found low positive predictive values (PPVs) for incident AVN (17–46%) but higher PPVs for prevalent or incident AVN (76–100%) compared to a gold standard of AVN diagnosis by comprehensive medical record review. The quality of the gold standard utilized, the generalizability of these results to non-VA populations, and the applicability of these algorithms to administrative databases where medical records are not available are currently unknown [9].

The primary objective of the current study was to develop and validate ICD-9 claims-based algorithms for the identification of AVN in an administrative database. We developed four algorithms of increasing stringency and determined the sensitivity, specificity, PPV, and positive likelihood ratio (LR^+^) of these claims-based algorithms to identify cases of AVN in a hospital imaging database.

## Methods

### Sample

Patients for this analysis were identified using the Partners HealthCare System Research Patient Data Registry (RPDR). The RPDR is a centralized clinical data registry from the Partners Health System hospitals, containing over 2.5 million patients and 550 million records including medical records with patient encounters, laboratory and radiology results [10, 11]. In addition, data from the Partners Health inpatient and outpatient billing system is directly downloaded into RPDR. We queried the RPDR database via an online Query Tool to identify subjects aged ≥18 years at the Brigham and Women’s Hospital (BWH) who underwent MRI for any indication between January 1, 2010 and June 1, 2011. MRIs of the upper and/or lower extremities were identified in RPDR using CPT codes 73,221, 73,222, 73,223, 73,721,73,722, or 73,723.

### AVN gold standard

Using RPDR, we downloaded a dataset of 8200 individuals who underwent MRI of the upper and/or lower extremities during the study period. We obtained radiologist reports from the medical record via use of the RPDR database which we subsequently ‘text mined’ for the following terms: “avascular necrosis”, “AVN”, “osteonecrosis”. We reviewed all MRI reports mentioning any of these terms, and those reports confirming the presence of AVN were considered to be an AVN case. We compared putative AVN cases identified by each algorithm to the gold standard of a clinical MRI report by a radiologist confirming AVN.

### ICD-9 Algorithms

Within this sample of patients who had all undergone MRIs, we examined the performance characteristics four algorithms ranging from least to most stringent (A–D) using ICD-9 codes for AVN (ICD-9, 733.4X).

- Algorithm A: ≥1 ICD-9 code for AVN at any time within the study period
- Algorithm B: ≥2 ICD-9 codes for AVN at least 7 days apart
- Algorithm C: ≥2 ICD-9 codes for AVN at least 30 days apart
- Algorithm D: ≥3 ICD-9 codes for AVN each at least 30 days apart

Only ICD-9 codes occurring within 6 months of MRI were considered.

### Demographics/co-morbidities

Age, sex, race, and co-morbidities (≥1 ICD-9 code within the 18-month study period for the following diagnoses: rheumatoid arthritis [ICD-9714.0], osteoarthritis [ICD-9715.X], systemic lupus erythematosus [ICD-9710.0] and human immunodeficiency virus [ICD-9042]) were extracted to describe AVN cases and non-cases.

### Statistical analysis

We estimated the prevalence of AVN among those with MRI as the proportion of confirmed cases of AVN on MRI report among all patients at BWH undergoing MRI of any upper and/or lower extremity joint for any indication. Baseline characteristics including demographic data and comorbidities were reported for AVN cases and non-cases.

To examine the performance of the four AVN case-finding algorithms, we determined the sensitivity, specificity, and PPV of claims-based algorithms for AVN compared to the gold standard [12]. We also determined LR^+^s, shown to be useful in validation studies as a tool for assessing the value of performing a diagnostic test by determining whether a test result usefully changes the a priori probability that a disease state exists. LR^+^ > 1 values argue in favor of the diagnosis of interest, with larger numbers being more suggestive of disease, whereas LR^+^ values from 0 to 1 argue against the diagnosis, with values closer to 0 being less likely disease.

We calculated sensitivity as the proportion of patients with AVN on MRI who also had the diagnosis by ICD-9 billing data. We calculated specificity as the proportion of patients without AVN on MRI who did not have AVN documented by ICD-9 claims. We calculated the PPV of ICD-9-based algorithms as the proportion of patients with AVN by the ICD-9-based algorithm who had the diagnosis of AVN confirmed by MRI [12]. We determined the positive likelihood ratios for each algorithm by dividing the sensitivity by one minus the specificity. We calculated 95% confidence intervals (95% CI) for sensitivity, specificity, and PPV using the normal approximation of the binomial distribution. We used validated formulae to calculate confidence intervals for positive likelihood ratios and AVN prevalence [13].

Analyses were conducted using SAS 9.3 software and positive likelihood ratio confidence intervals were calculated using R 3.2.5 software.

## Results

Study cohort (Table 1): Among 8200 patients who underwent MRI of the upper and/or lower extremities during the 18-month study period, 83 cases of AVN were identified on MRI, yielding a prevalence of 1.0% [95%CI 0.78–1.22]) The mean age of patients with AVN on MRI was 50.4 years (standard deviation [SD] 15.1), with 60.2% of these patients being female. While the proportion of subjects who were White, Hispanic, and Asian did not differ substantially among patients with AVN on MRI compared to those without AVN on MRI, there was a higher proportion of Blacks (15.7% versus 6.7%) among the AVN cases than non-cases. Furthermore, patients with AVN on MRI had a higher prevalence of rheumatoid arthritis, systemic lupus erythematosus, and osteoarthritis (Table 1).

**Table 1:** Baseline characteristics of 8200 study subjects based on evidence of AVN by MRI

	AVN Cases (evidence of AVN on MRI) *n* = 83	Non-Cases (without AVN on MRI) *n* = 8117
Mean age ± SD	50.4 ± 15.1	52.4 ± 15.4
Females, *n* (%)	50 (60.2)	4541 (55.9)
Race, *n* %		
White	57 (68.7)	5975 (73.6)
Black	13 (15.7)	542 (6.7)
Hispanic	4 (4.8)	458 (5.6)
Asian	4 (4.8)	145 (1.8)
Other	0 (0)	64 (0.8)
Not Recorded	5 (6.0)	933 (11.5)
Co-morbidities^a^, *n* (%)		
Rheumatoid Arthritis	5 (6.0)	205 (2.5)
Osteoarthritis	35 (42.2)	2410 (29.7)
Systemic Lupus Erythematosus	8 (9.6)	29 (0.4)
HIV	0 (0)	16 (0.2)

^a^Defined as ≥1 ICD-9 code within the 18-month study period using the following codes: Rheumatoid Arthritis (ICD-9714.0), Osteoarthritis (ICD-9715.X), Systemic Lupus Erythematosus (ICD-9710.0), HIV (ICD-9042).

Performance characteristics for the four claims-based algorithms are shown in Table 2. As the algorithm stringency increased, specificity increased (from 99.6% for Algorithm A to 99.9% for Algorithm D) at the expense of diminished sensitivity (from 81.9% to 44.6%). Along with specificity, the PPVs increased from 66% to 82% with greater algorithm stringency. Algorithm A had the highest sensitivity at 81.9% (95% CI 72.0–89.5%), with a high LR+ of 190 (95% CI 134.5–268.5%). However, the specificity and PPV were lowest for Algorithm A. The specificities and PPVs were similar for Algorithms B and D (99.9% versus 99.9% and 81.4% versus 82.2%, respectively), but the sensitivity of Algorithm B was considerably higher than that of Algorithm D (57.8% versus 44.6%). In our cohort with relatively low prevalence of AVN, Algorithm B demonstrated moderately high sensitivity (57.8%), high specificity (99.9%), high positive likelihood ratio (426.7), and high PPV (81.4%). Table 3 depicts the 2 × 2 table used to calculate the sensitivity, specificity, and PPV for Algorithm B. Compared to Algorithm D, Algorithm C had a similar sensitivity and specificity but had a lower PPV (78.7% versus 82.2%) and positive likelihood ratio (361.8 versus 452.3).

**Table 2:** Performance characteristics of ICD-9-based algorithms for the diagnosis of avascular necrosis (AVN)

Algorithms	Sensitivity %, (95% CI)	Specificity %, (95% CI)	PPV %, (95% CI)	LR^+^ (95% CI)
A: ≥1 ICD-9 code for AVN	81.9 (72.0–89.5)	99.6 (99.4–99.7)	66.0 (56.0–75.1)	190.0 (134.5–268.5)
B: ≥2 ICD-9 codes for AVN at least 7 days apart	57.8 (46.5–68.6)	99.9 (99.8–99.9)	81.4 (69.1–90.3)	426.7 (229.9–792.1)
C: ≥2 ICD-9 codes for AVN at least 30 days apart	44.6 (33.7–55.9)	99.9 (99.8–99.9)	78.7 (64.3–89.3)	361.8 (186.2–703.1)
D: ≥3 ICD-9 codes for AVN at least 30 days apart	44.6 (33.7–55.9)	99.9 (99.8–100.0)	82.2 (67.9–92.0)	452.3 (217.3–941.4)

All ICD-9 codes occurred within 6 months of MRI.

Abbreviations: *ICD-9* International Classification of Diseases, 9th version, *CI* Confidence Interval; *PPV* Positive Predictive Value, *LR* ^*+*^ Positive Likelihood Ratio

The sensitivity of algorithm C is actually 44.578 and specificity of 99.877, whereas the sensitivity of algorithm D is 44.578 and the specificity is 99.901, accounting for the discrepancy in the observed likelihood ratios of algorithm C and D.

**Table 3:** 2 × 2 table depicting results for Algorithm B^a^, optimal for use when medical record review is not possible^a^

		MRI Result (Gold standard)		
Algorithm B		AVN (+)	AVN (−)	Total
	Algorithm (+) for AVN	48	11	59
	Algorithm (−) for AVN	35	8106	8141
	Total	83	8117	8200

^a^Algorithm B: Defined as ≥2 ICD-9 codes for AVN at least 7 days apart within 6 months of MRI; Sensitivity = 57.8% (95% CI 46.5–68.6), PPV = 81.4% (95% CI 69.1–90.3).

## Discussion

AVN cases identified according to algorithms B and D (both with PPV > 80%) are most likely to be true cases by our gold standard definition. Thus, researchers aiming to identify a highly specific cohort of AVN patients for use in epidemiologic studies may use either of these algorithms with the understanding that >80% of subjects identified by these algorithms are likely to have true AVN compared to an MRI gold standard. Furthermore, the very high positive likelihood ratios (>100) demonstrate a large increase in the post-test probability of identifying AVN with use of any of these four algorithms, particularly algorithms B or D (LR+ for both >400). Therefore, given a pre-test probability of 1%, with a likelihood ratio of >400, the post-test odds of finding an AVN case using Algorithms B or D would be increased at least four-fold.

Administrative data are being increasingly used in rheumatic disease health services research and are particularly helpful in order to study rare diseases such as AVN as they provide large study populations and are unaffected by recall [14, 15]. However, confirming the accuracy of case ascertainment algorithms through a validation study is an important step in reducing misclassification error-- a potential bias arising in research conducted with administrative data. Furthermore, accurate identification of AVN cases will allow for better disease surveillance including improved estimates of prevalence and incidence. For example, an algorithm with 100% sensitivity captures all AVN cases, but may also capture false positives; whereas, an algorithm with 50% sensitivity will identify fewer AVN cases and thus underestimate disease prevalence and incidence. Therefore, from a research perspective, selection of an appropriate and efficient ICD code based algorithm for accurate identification of AVN cases in administrative data should first aim to optimize PPV and LR+ to reduce misclassification and then attempt to minimize missed cases by maximizing sensitivity.

In this study, the high sensitivity of Algorithm A indicates that the majority of true cases are identified, with few false negatives; however the PPV of this algorithm is the lowest. Therefore, Algorithm A may be useful as a screening tool for AVN in situations where further medical record review is feasible in order to rule out false positives. Algorithm B, requiring ≥2 ICD-9 codes at least 7 days apart, had a high PPV and higher sensitivity compared to algorithms C or D, and is useful when further medical record review is not feasible, although misclassification may still occur. Algorithm D had the highest PPV and LR^+^ compared to all of the other algorithms, but had a substantially lower sensitivity than Algorithm B (44.6% versus 57.8%, respectively). Given its high specificity, PPV, and LR^+^, Algorithm D can similarly be used in situations where further medical record review is not feasible. However given its lower sensitivity compared to Algorithm B, this algorithm would identify fewer cases. Compared to Algorithms B or D, Algorithm C does not appear to confer an advantage given its lower sensitivity (compared to Algorithm B), and lower LR^+^ and PPV (compared to Algorithms B and D). Therefore, in our study population with a gold standard MRI-diagnosed AVN prevalence of 1.0%, use of Algorithm B slightly underestimates prevalence (0.7%), whereas Algorithm A overestimates prevalence (2.5%). As the misclassification of cases (i.e. false positive rate) using Algorithm A is higher than Algorithm B (0.34 vs. 0.19), Algorithm B would be optimal when confirmatory medical chart review is not feasible.

Various explanations exist for the observed decrease in algorithm sensitivity with increasing stringency. Due to the tertiary care medical center population analyzed, patients may have been referred for a single visit and MRI, but returned to their primary care institution for further management, resulting in a true case of AVN with a single ICD-9 code for AVN in our database. In addition, patients who received an AVN diagnosis immediately prior to death or those who were lost to follow-up may have only received one ICD-9 code for AVN at the institution. Given that patients with AVN may be medically complex with multiple co-morbidities, providers may not prioritize billing for AVN beyond the initial visit or diagnosis. Furthermore, if the AVN diagnosis is considered to be mild or asymptomatic, requiring only conservative management and no further referral to specialists, providers may similarly not bill for AVN beyond the initial diagnosis. It is possible then, that cases identified by more stringent algorithms are actually more severe cases of AVN.

To our knowledge, only one prior study has evaluated the use of ICD-9 based algorithms for the identification of cases of prevalent and incident AVN in a large health care database [9]. In this study from a large US Department of Veterans Affairs (VA) database which utilized ICD-9 billing codes for AVN and other disease states including osteoarthritis, the PPVs for incident AVN remained low (17–46%) despite the availability of complete medical record data of VA-provided health care for each veteran. Although PPVs for identifying prevalent or incident AVN in the VA database were higher than those demonstrated in our study (ranging from 76 to 100%), this may be related to the use of comprehensive clinical data--including clinic notes, discharge summaries, radiology reports, and actual radiographs—as the “gold standard” for case confirmation. However, use of comprehensive medical record review as the gold standard, which did not necessarily require evidence of AVN on MRI, may rely more heavily on alternative measures of AVN and may thus reduce the accuracy of the diagnosis. In addition, the generalizability of the sensitivity and specificity of these algorithms to non-VA cohorts and other health care settings is limited by the known male predominance of the VA study (92%), potential differences in patient preference for treatment and provider practices, and potentially higher rates of risk factors such as alcohol abuse, early osteoarthritis and history of trauma to joints [16–18]. Furthermore, the VA study did not provide data on sensitivity, specificity, or positive likelihood ratios of algorithms tested, which may be useful for researchers attempting to utilize these algorithms in different populations. In our study, we did not attempt to distinguish between incident versus prevalent AVN given the inherent limitation to using diagnosis codes for prediction of incident disease due to the inability to confirm relevant details such as the date of symptom onset and duration, previous imaging performed elsewhere, and the diagnosis date.

Results of this study should be viewed within some limitations. Our gold standard definition required evidence of AVN on MRI; therefore, cases of asymptomatic or early AVN that did not prompt MRI were not included in the analysis. This suggests that use of our case-finding method may identify more severe and/or symptomatic cases, while excluding milder cases of AVN. Thus, the possibility of a spectrum bias in our study exists, such that in a sample with less symptomatic AVN, the sensitivity of our algorithms may be lower [19]. Additionally, as our study population derived from a single, tertiary care level academic center located in northeastern United States, varying provider practices and patient characteristics may affect the performance characteristics of the algorithms in other healthcare or geographic settings. Therefore, the sensitivity and specificity should be tested further in other independent patient samples to establish their generalizability. Furthermore, given the adoption of ICD-10 in the United States, future work replicating our algorithms using ICD-10 codes may prove useful. We suspect that performance characteristics using ICD-10 AVN codes will be similar or better, given the increased categorization and granularity of ICD-10 AVN codes including specific joint location, etiology (i.e. idiopathic vs. secondary causes including trauma, drugs, hemoglobinopathies), and exclusion of osteonecrosis of the jaw.

## Conclusions

Although administrative data are imperfect sources of clinical information, they are particularly useful for large-scale epidemiologic research focusing on risk factors and outcomes for rare diseases. In this study we developed and validated an ICD-9 claims-based algorithm for identifying AVN cases among individuals undergoing MRI of the upper and lower extremities using a centralized patient registry from a U.S. academic medical center. The positive predictive values of the algorithms were moderately high, ranging from 66 to 82%, and associated with very high specificity (>99%). Our study results demonstrate that algorithms utilizing billing codes for AVN provide an efficient way to identify AVN cases in administrative data, which is a novel and relevant finding for future AVN research studies. Further research is necessary to determine whether the performance characteristics will differ in distinct populations such as systemic lupus erythematosus or orthopedic cohorts, or in other geographic settings.

## Abbreviations

AVN: Avascular necrosis
BWH: Brigham and women’s hospital
ICD-10: International classification of diseases-10
ICD-9: International classification of diseases-9
LR +: Positive likelihood ratio
MRI: Magnetic resonance imaging
PPV: Positive predictive value
RPDR: Research patient data registry
SD: Standard deviation
